# Rational Use of Elimination Diets in Infants and Toddlers: Navigating the Overlap Between Disorders of Gut–Brain Interaction (DGBI) and Food Allergy—A Narrative Review

**DOI:** 10.3390/nu18193211

**Published:** 2026-09-29

**Authors:** Anna Stochel-Gaudyn, Agnieszka Kozioł-Kozakowska, Kinga Kowalska-Duplaga

**Affiliations:** Department of Pediatrics, Gastroenterology and Nutrition, Jagiellonian University Medical College, 31-008 Cracow, Poland; agnieszka.koziol-kozakowska@uj.edu.pl (A.K.-K.); kinga.kowalska-duplaga@uj.edu.pl (K.K.-D.)

**Keywords:** allergy, elimination diet, gut–brain interaction, functional disorders, nutrition

## Abstract

Disorders of gut–brain interaction (DGBI) are the most common cause of gastrointestinal symptoms in infants and toddlers. The overall prevalence in the pediatric population aged 0–48 months is approximately one in four children. DGBI symptoms such as excessive crying, regurgitation, constipation or altered bowel movements can often mimic or overlap with food allergy (FA) manifestations, especially non-IgE-mediated allergy, thus posing a diagnostic and therapeutic challenge. As a consequence, prolonged empiric elimination diets are often initiated in DGBI management despite limited evidence-based data. Although temporary clinical improvement may be observed and suggestive of FA, failure to perform allergen reintroduction can lead to unnecessary elimination, serious nutritional deficiencies, compromised development of oral skills, neophobia and reduced quality of life for both the child and its caregivers. It can also increase healthcare costs. The aim of this narrative review is to identify current evidence supporting the use of elimination diets in the most common DGBI in infants and toddlers, which include infantile colic, regurgitation, dyschezia and functional constipation, as well as functional diarrhea. We also emphasize distinguishing physiological and functional symptoms from allergy symptoms, as well as identifying clinical situations where both conditions can co-exist, thus justifying diagnostic elimination diets. Finally, we highlight both the benefits and potential harms of dietary restrictions in order to support an accurate diagnostic approach and optimize nutritional management.

## 1. Introduction

Gastrointestinal and feeding symptoms are very common in infants and small children, often causing worry and stress both in parents and physicians, and can lead to unnecessary elimination diets early on in a child’s life. This age group is especially vulnerable to even short-term dietary restrictions, as they can compromise a child’s proper weight gain, growth, feeding habits or motor development. Following long-term elimination diets can also increase parent stress and family financial burden.

Symptoms such as excessive crying, regurgitation, bloating, changes in stooling habits or abdominal discomfort are nonspecific and can be present in both disorders of gut–brain interaction (DGBI) and food allergy (FA), especially in very young children. On top of that, DGBI and FA may both co-exist in the same patient, further complicating clinical assessment. However, the prevalence of these two conditions varies significantly. While DGBI are common in infants and toddlers, with a prevalence of approximately 22% (5.8–40%) depending on the study methodology, FA are significantly less common, with a prevalence of approximately 5–8%, whereas challenge-confirmed FA account for only 2–4%. Cow’s milk allergy (CMA) remains the most frequent food allergy during infancy, while hen’s eggs, wheat, soy and peanuts are also common triggers [1,2,3,4]. It is important to highlight that symptom improvement after elimination diet introduction, without food challenge or allergen reintroduction, does not prove causality and thus creates a significant diagnostic gray zone in early childhood. This clinical uncertainty, together with the absence of specific biomarkers in DGBI and non-IgE-mediated FA, often contributes to prolonged and widespread food removal in infants and toddlers without any sound evidence [5].

Elimination diets may be necessary in cases highly suspicious of FA, such as children with alarm features including failure to thrive and bleeding from the gastrointestinal tract, or children presenting with symptoms from additional systems, like the skin or pulmonary tract, as well as children who had a positive oral food challenge or failed to pass allergen reintroduction. There is little evidence in the literature, though, supporting the use of elimination diets in infants and toddlers with DBGI. This is why it is vital to underline that unjustified and prolonged restrictions can lead to nutritional deficiencies and compromise a child’s healthy growth and development. On top of that, delayed allergen reintroduction may reduce opportunities for oral tolerance development and maintenance [6]. Current guidelines for FA management in this age group recommend targeted and short-term food elimination followed by reintroduction and clinical assessment, allowing identification of children who will benefit from restrictions. Such an intervention should always be supported by adequate nutritional counseling provided by a clinical dietitian and regular medical assessment [7,8]. Nevertheless, a considerably variable approach is observed in clinical practice, especially in infants and toddlers who improve after implementing restrictions or in those presenting with overlapping symptoms suggestive of both DBGI and FA.

In this narrative review, we aim to summarize current evidence on the rational use of elimination diets in infants and toddlers with DGBI, with a special emphasis on the symptom overlap between DBGI and FA in this age group, which often leads to unnecessary and prolonged food restrictions. We also aim to address potential risks connected to elimination diets, such as nutritional deficiencies, failure to thrive, compromised motor development, food avoidance, reduced quality of life and financial burden. Finally, we aim to propose a practical framework for managing and differentiating these conditions to support evidence-based clinical decision-making.

## 2. Materials and Methods

This article is a narrative review aimed at summarizing current evidence regarding the rationale for using elimination diets in the most common DGBI, such as infantile colic, regurgitation, dyschezia and functional constipation, as well as functional diarrhea in infants and toddlers. We address the problem of distinguishing physiological and functional symptoms from allergy symptoms, as well as identifying clinical situations where both conditions can co-exist, justifying diagnostic elimination diets. We also evaluate the evidence on both the benefits and potential harms of dietary restrictions in this age group.

A literature search was conducted in the databases PubMed, Scopus, Web of Science and Google Scholar. Suitable entries published between 2015 and 2026 were screened for topics reflecting the need for straight-forward clinical algorithms that would be useful in everyday practice. Three older publications, published between 2005 and 2015, were included because they reported the natural history of specific FA. To ensure comprehensive coverage of the topic, clinical practice guidelines, expert consensus statements, systematic reviews, meta-analyses and observational studies were included.

Studies were excluded if they focused exclusively on adult populations, examined pharmacological treatment of DGBI without dietary intervention, consisted only of conference abstracts without full text, or lacked sufficient methodological quality based on the authors’ judgment.

Due to the narrative nature of this review, quantitative meta-analysis and formal risk of bias assessment were not conducted. Instead, emphasis was placed on qualitative synthesis and comparative interpretation of available evidence, highlighting gaps in knowledge, variability in diagnostic and treatment protocols, and practical difficulties encountered in infants and toddlers with DGBI.

## 3. Disorders of Gut–Brain Interaction in Early Childhood

Disorders of gut–brain interaction (DGBI), formerly known as functional gastrointestinal disorders, represent a significant clinical challenge in pediatric medicine, affecting approximately one in four children worldwide [9,10]. These conditions are characterized by chronic or recurrent gastrointestinal symptoms that cannot be explained by structural or biochemical abnormalities but instead arise from dysregulation of the complex bidirectional communication between the central nervous system and the enteric nervous system; they are classified according to Rome V Criteria. The parallel development of the gut, the brain, and the microbiome makes early childhood particularly critical for the emergence of DGBI. Disruptions during this stage, whether through medical interventions, psychological stressors, or environmental factors, can alter the trajectory of the microbiota–gut–brain axis and predispose individuals to long-term gastrointestinal dysfunction [11,12]. Understanding the nuances of these early-life DGBI is essential for providing timely diagnosis and avoiding unnecessary, invasive medical evaluations, unjustified diets or attempts at treatment [13]. The prevalence varies depending on the type of condition, age and geographical region. In a cohort of three-year-old children, functional constipation was identified as the most prevalent DGBI at 20.4%, followed by infant colic/distress syndrome at 4.6%, infantile regurgitation at 2.8%, and infant dyschezia at 1.6%. Interestingly, sex appears to be a significant factor even in this young population, with some evidence suggesting a higher overall prevalence of DGBI in girls than in boys (28.1% vs. 20.7%) [10]. Furthermore, there is a recognized overlap, where approximately 4% of children may fulfil the criteria for multiple DGBI diagnoses simultaneously. Some reports also point to regional variations. Certain studies carried out in the United States suggest that as many as a quarter of all children meet the DGBI criteria, whilst cohort studies in Italy and Vietnam indicate a prevalence ranging from 10 to 21% [14,15].

Infantile regurgitation is a specific early childhood DGBI. This condition is characterized by the effortless return of gastric contents into the mouth. It is often considered a normal physiological occurrence in early infancy that resolves as the child matures. According to current Rome V Criteria, the diagnosis applies to healthy infants between 3 weeks and 12 months of age who experience regurgitation at least two times per day for a minimum of three weeks. Crucially, the diagnosis requires the absence of “red flag” symptoms such as retching, hematemesis, aspiration, apnea, failure to thrive, or feeding/swallowing difficulties. While common in the first six months of life, symptoms typically peak and then decline significantly as the infant reaches term-equivalent age and begins sitting up and consuming solid foods.

Infant dyschezia is a frequently misunderstood condition that often causes significant caregiver anxiety despite its benign nature. Dyschezia is caused by a lack of coordination between increased abdominal pressure and the relaxation of the pelvic floor muscles. According to Rome V Criteria, it is diagnosed in infants younger than 9 months who exhibit at least 10 min of straining and crying before the successful or unsuccessful passage of soft stools. Supportive criteria include screaming, extreme reddening of the face, and visible effort during these episodes. It is worth emphasizing that the condition is self-limited; it resolves as the infant “learns” to relax the pelvic floor during defecation. Caregivers are strongly advised to avoid rectal stimulation, as this can interfere with the child’s natural learning process and may condition the infant to wait for external stimuli before defecating [16].

In the transition to Rome V Criteria, the term “infantile colic” was replaced by “Infant Distress Syndrome” (IDS) to move away from the unproven implication that the pain originates solely in the colon. This new nomenclature emphasizes the visible distress of the infant and the involvement of the gut–brain axis. Clinical criteria for IDS include an infant under 5 months of age at symptom onset who experiences recurrent and prolonged periods of crying, fussing, or irritability reported by caregivers. These episodes occur without an obvious cause and cannot be prevented or resolved by the caregiver. For research purposes, the “Rule of Three” is often applied: crying lasting more than 3 h per day for at least 3 days per week. Symptoms typically peak between 1 and 2 months of age [16]. IDS is believed to be multifactorial, involving gut dysbiosis (increased Proteobacteria, decreased Bifidobacteria), neurologic immaturity (greater reactivity to sensory stimuli), and psychosocial factors such as maternal anxiety. In some studies, exclusive breastfeeding for at least three months has been shown to be a significant protective factor against the development of IDS, but the data are inconclusive to draw strong conclusions [17].

The most common condition within the DGBI group encountered in specialist pediatric care is functional constipation (FC). It often represents a chronic condition that can persist from early childhood to adolescence. The diagnosis requires at least one month of symptoms, including two or fewer defecations per week, history of excessive stool retention or retentive posturing, and history of painful or hard bowel movements (Bristol Stool Form Scale type 1 or 2). In infants and children who are not yet toilet-trained, the criteria emphasize retentive posturing and straining, which parents often misinterpret as effort to pass stool when it is actually an effort to withhold it. FC typically begins with a triggering event—such as a painful bowel movement—which leads the child to develop a universal instinct to avoid defecation. This results in a “vicious cycle”: the withheld stool remains in the rectum, where water is absorbed, making the mass harder and larger, eventually leading to further painful defecation, overflow fecal incontinence, and a loss of the normal urge to defecate. Low fiber and fruit consumption, family history, and a lack of toilet training are associated with an increased risk. Conversely, exclusive breastfeeding for at least six months appears to provide significant protection against FC [10,16].

It is vital to highlight that the presence of gastrointestinal symptoms during infancy and early childhood does not necessarily indicate an underlying organic disease, as many may reflect normal developmental physiology and age-related variation. In otherwise thriving infants, manifestations such as regurgitation, crying, abdominal distension, altered stooling patterns, or straining are common and often self-limiting. When symptoms fulfil Rome V diagnostic criteria and alarm features are absent, they may be classified as disorders of gut–brain interaction (DGBI). Conversely, the presence of alarm features or other clinical indicators—such as faltering growth, persistent vomiting, gastrointestinal bleeding, chronic diarrhea, marked eczema, or reproducible symptoms following ingestion of a specific food—should prompt evaluation for an underlying organic disorder, including an FA. The alarm signs indicating organic disorders are summarized in Table 1.

## 4. Food Allergy in Infants and Toddlers

A food allergy (FA) in infants and young children is a major clinical concern because it often presents early in life, affects nutrition and growth, and may be difficult to recognize on clinical grounds alone. A food allergy is defined as an adverse health effect arising from a specific, reproducible immune response following exposure to a particular dietary protein. These reactions are broadly classified into three categories based on the underlying immunological pathway: IgE-mediated, non-IgE-mediated (primarily cell-mediated), and mixed mechanisms. While IgE-mediated reactions are characterized by immediate onset (within minutes to hours), non-IgE-mediated reactions are typically delayed: from several hours up to days or weeks after ingestion. The prevalence of food allergies in the pediatric population is rising, affecting approximately 3% to 10% of children globally [8]. While parental perception of FAs is often higher and reaches 10%, positive oral food challenge (OFC) or planned allergen reintroduction, the “gold standard” for FA diagnosis, confirms the condition in less than 1% of suspected infants. Data from the EuroPrevall birth cohort study reported an overall incidence of challenge-proven cow’s milk allergy (CMA) of 0.54% across Europe, though national figures vary from <0.3% to 1%. Interestingly, in exclusively breastfed infants, the incidence is remarkably lower and estimated at 0.4% to 0.5%, with IgE-mediated reactions occurring in fewer than one in 1000 cases [18,19,20].

CMA is the most common FA in children under three years of age. Other major food allergens include hen’s eggs, peanuts and tree nuts, wheat, soy, fish, and shellfish. Although the distribution of specific food allergens varies across countries due to differences in dietary habits, food labeling practices, and the timing of allergenic food introduction, a similar pattern of FA is consistently observed in pediatric populations worldwide. Egg allergy, like CMA, is particularly common during the first years of life. Although it resolves spontaneously in many children, it remains one of the leading causes of IgE-mediated allergic reactions in early childhood. The overall prevalence of egg allergy in children is estimated at approximately 1.6%. However, marked geographic differences have been reported, with the Australian HealthNuts study documenting a raw egg allergy prevalence of 8.9% among infants, compared with approximately 1.0% in Canadian children [21,22].

Peanut, tree nut, fish, and shellfish allergies are less common in the first year of life but are clinically important because they are more often associated with systemic reactions and a more persistent disease course. The reported prevalence of peanut and tree nut allergies varies across populations, affecting approximately 2.2% of Canadian children and 3.0% of Australian infants. In Europe, the cumulative lifetime prevalence is estimated at approximately 1.5%, whereas in the United States, the cumulative prevalence among infants has been reported to range between 0.67% and 0.92%. By contrast, the prevalence of allergies to fish and shellfish is very low, ranging from 0.05% to 0.9% [23,24]. The frequency of these allergies changes as children age. By the age of 16, most children gain tolerance to allergens like cow’s milk and eggs, with 79% for CMA and 68% for egg allergy. In contrast, allergies to peanut, tree nuts, and seafood are often lifelong, with only about 20% of children gaining tolerance [25,26,27,28].

The clinical presentation of FA in infants and young children is heterogeneous and depends largely on the underlying immune mechanism. IgE-mediated food allergies usually cause rapid symptoms, often within minutes to hours after ingestion, although the timing may sometimes be less clear in real-world settings. Skin symptoms are among the most visible signs and often prompt referral, but gastrointestinal and respiratory symptoms may predominate in some children. By contrast, non-IgE-mediated food allergies often present with delayed and more chronic symptoms, which makes clinical recognition more challenging. In infants, manifestations may be subtle and nonspecific, such as recurrent vomiting, feeding refusal, irritability, poor sleep, or a transient flare of eczema after exposure to a suspected food. Specific allergic reactions presenting from the gastrointestinal system include conditions such as food protein-induced allergic proctocolitis (FPIAP), food protein-induced enterocolitis syndrome (FPIES), food protein-induced enteropathy (FPE) or eosinophilic gastrointestinal disease. The immunological mechanisms of these conditions are heterogeneous and incompletely understood, and their presentations may be immediate, like in FPIES, or postponed, like in FPIAP or FPE.

Immediate, non-immediate and delayed reactions and the most common clinical manifestations are summarized in Table 2.

The diagnostic process in pediatric FA begins with a careful history because the probability of an allergy depends strongly on the pattern, timing, reproducibility, and severity of symptoms after exposure to a specific food. The clinical history regarding allergies is the first and most important step. It should cover the age at which the first symptoms appeared, the nature and frequency of the symptoms, and the time between ingestion and onset of the reaction. Family history of atopy may also be an indicator. In the case of infants, information regarding feeding methods (breastfed or formula-fed) and growth parameters (percentile charts) is essential. A physical examination can provide supportive but rarely definitive information. In IgE-mediated diseases, cutaneous findings such as urticaria, flushing, eczema exacerbation, or angioedema may be present, and respiratory signs may include wheezing or upper-airway symptoms. In mixed etiology or non-IgE-mediated disorders, an examination may reveal poor growth, pallor, abdominal distension, signs of feeding difficulty, or manifestations of atopic dermatitis. However, normal examination findings do not exclude FA [7,8].

Laboratory tests are an important adjunct to history, but they must be used selectively and interpreted in the context of a clinical picture. Skin prick tests and serum food-specific IgE measurement are the most widely used tests for a suspected IgE-mediated allergy. Both detect sensitization rather than clinical reactivity, which means that a positive result does not automatically confirm an FA [8]. In non-IgE-mediated food allergies, standard IgE-based tests are often negative or uninformative. This creates a major diagnostic difficulty because symptoms may be real and clinically significant even when serum IgE and skin tests are normal. There is no single validated biomarker that can reliably confirm most non-IgE-mediated FA Patch testing has been explored in some settings, particularly for delayed cutaneous or gastrointestinal reactions, but it is not sufficiently standardized for routine diagnosis in pediatric practice. Planned oral allergen reintroduction remains the reference standard for confirming or excluding FA, particularly when the diagnosis is uncertain. The interpretation of symptoms after elimination and reintroduction is one of the central difficulties in pediatric FA diagnosis. Improvement during elimination may support the diagnosis, but it is not specific because symptoms such as vomiting, eczema, or irritability may fluctuate naturally over time or be a presentation of a DGBI.

## 5. Clinical Overlap Between Disorders of Gut–Brain Interaction and Food Allergy

The overlap between FA and DGBI such as regurgitation, infantile distress syndrome, previously known as infantile colic, functional diarrhea or constipation, contributes substantially to diagnostic uncertainty because all of them may present with gastrointestinal symptoms related to food intake. Symptoms such as excessive crying, bloating, altered bowel movements or feeding aversion, although stressful for caregivers and physicians, are non-specific manifestations of gastrointestinal dysfunction and cannot by themselves be interpreted as FA. The greatest diagnostic challenge is therefore to distinguish true immune-mediated disease from coincidental symptoms or non-allergic intolerance. Overdiagnosis is a well-recognized problem because unnecessary elimination diets may impair nutrition, burden families, and delay the identification of the true cause of symptoms. Underdiagnosis is also common, especially in non-IgE-mediated diseases, where the absence of immediate symptoms and the lack of a reliable laboratory marker often lead to missed or delayed diagnosis [29,30,31].

A detailed feeding history remains an essential part of evaluating suspected DGBI or FA in infants and toddlers, as improper eating practices may be the cause or may increase gastrointestinal symptoms attributed to FA. Information such as type of feeding (breastfeeding, formula feeding, complementary feeding); frequency, volume and duration of feeds; preparation of formula; bottle teat flow or latching and milk transfer during breastfeeding; feeding position; burping practices; and caregiver responsiveness to the child’s needs are crucial in assessing potential malpractices. Feeding large volumes, especially over a short period of time and in short intervals, and improper positioning while breastfeeding or horizontal positioning while bottle feeding increase gastric distention and intragastric pressure, leading to exacerbation of regurgitation, abdominal discomfort, crying and irritability in the child. All of these symptoms may be falsely attributed to FA [32,33]. Growth curves should be assessed alongside eating habits. Rapid weight gain, especially disproportionate to linear growth, may be suggestive of excessive caloric and volume intake. Excessive feeding, in addition to causing gastric distention and increased regurgitation, can exceed an infant’s digestive and absorptive capacity, especially for carbohydrates such as lactose, thus increasing the delivery of fermentable substrates to the colon. As a result, escalated luminal fermentation, gas production and osmotic effects may be observed, contributing to abdominal distention, excessive crying and altered stooling patterns, while potentially interacting with the developing gut microbiota [34,35,36].

Initial management in children suspected of DGBI, especially in the absence of alarm features (summarized in Table 1), such as rectal bleeding, atopic manifestations, persistent diarrhea, vomiting or impaired growth, should concentrate on feeding habit modification. Feeding volume and frequency should be adjusted while maintaining the child’s daily caloric intake, with avoidance of excessive volume and long feeding intervals. Attention should be paid to feeding position, pacing, teat flow, burping and caretaker responsiveness to the child’s needs. If regurgitation is the predominant symptom, thickeners or specialized thickened anti-regurgitation formulas may be considered, followed by symptom and stooling pattern assessments, together with assessments of feeding tolerance and the child’s growth. Improvement after these conservative interventions, although potentially incomplete, supports the diagnosis of DGBI and prevents unnecessary elimination diets. On the other hand, if symptoms persist despite feeding optimization, especially in combination with the presence of alarm features, alternative diagnoses should be taken into consideration, such as FA. A targeted short diagnostic elimination diet followed by planned allergen reintroduction should be implemented [7,8,37].

## 6. Elimination Diets: When Are They Justified?

Empirical elimination diets are frequently prescribed to infants presenting with gastrointestinal symptoms, despite the fact that most symptoms are attributable to physiological conditions or DGBI rather than FA. Current international guidelines consistently emphasize that dietary elimination should not be prescribed empirically but rather be reserved for carefully selected clinical situations in which a food allergy is strongly suspected or confirmed. In particular, recent position papers and guidelines from the European Society for Pediatric Gastroenterology, Hepatology and Nutrition (ESPGHAN), the World Allergy Organization (WAO DRACMA), and the European Academy of Allergy and Clinical Immunology (EAACI) recommend a short-term (typically 2–4 weeks) diagnostic elimination followed by planned allergen reintroduction or oral food challenge to confirm the diagnosis of a FA. The duration of an elimination diet and diagnostic strategy may depend on the type of delayed reaction and suspected condition. For instance, in some specific conditions like FPE, the diagnostic elimination may have to last up to 8 weeks to observe symptom improvement. A summary of guidelines is presented in Table 3 [7,8,38,39].

Although international societies uniformly recommend elimination diets in the diagnosis and management of confirmed or suspected FA, they consistently discourage their empirical use in infants presenting with isolated gastrointestinal symptoms. This cautious approach reflects the high prevalence of physiological conditions and DGBI during infancy, the nonspecific nature of gastrointestinal symptoms, and the limited diagnostic value of symptom improvement following dietary elimination alone. In the absence of an oral food challenge or planned allergen reintroduction, clinical improvement cannot be regarded as proof of an FA because spontaneous symptom resolution and the fluctuating natural course of DGBI are common during early childhood. Consequently, current guidelines emphasize that elimination diets should be viewed primarily as a diagnostic tool rather than an empirical therapeutic intervention. The available evidence supporting elimination diets in the most common DGBI is summarized in Table 4.

The available evidence reveals a clear discrepancy between the frequent use of elimination diets in clinical practice and the available evidence supporting their efficacy in infants with DGBI. While current international guidelines consistently recommend elimination diets as part of the diagnostic and therapeutic management of confirmed or strongly suspected FA, evidence supporting their use in isolated DGBI remains limited and inconsistent, or is lacking altogether, depending on the specific disorder. Consequently, routine empirical elimination diets should not be recommended for infants presenting with isolated functional gastrointestinal symptoms.

A rational approach to elimination diets therefore requires careful patient selection, recognition of alarm features suggestive of FA, and strict adherence to evidence-based recommendations. Whenever a diagnostic elimination diet is initiated, it should be time-limited, nutritionally adequate, and always followed by planned allergen reintroduction or an oral food challenge to confirm or exclude FA. Such an approach minimizes unnecessary dietary restrictions while reducing the risk of overdiagnosis and overtreatment. At the same time, the limited available evidence highlights the need for well-designed randomized controlled trials evaluating dietary interventions in infants with DGBI. The nutritional and psychosocial consequences associated with unnecessary elimination diets are discussed in the following section.

## 7. Nutritional Safety: Risks of Unnecessary Elimination Diets

Every elimination diet ordered without proper medical and dietary counseling, especially in rapidly growing and developing infants and toddlers, raises questions about nutritional safety. As CMA seems to be the most common FA in this age group, an elimination diet without cow’s milk protein is usually the first to be implemented. Cow’s milk and dairy products are an important source of energy, high-quality protein, fat, calcium, phosphorus, iodine, zinc, riboflavin and vitamins D and B12. Therefore, their elimination without adequate nutritional replacement, and when indicated, appropriate supplementation, may result in insufficient nutrient intake and compromise growth and development. From a dietary perspective, nutritional safety depends not only on supplementation but also on adequate replacement of excluded food, with age-appropriate, nutritionally equivalent alternatives, taking into account a child’s energy and nutrient requirements, growth trajectory and overall dietary pattern. In exclusively breastfed infants, elimination is undertaken by mothers while maintaining breastfeeding; thus, dietary counseling and supplementation of calcium and vitamin D should include mothers. In formula-fed infants, standard cow’s milk formulas should be replaced by specialized formulas such as extensively hydrolyzed formula (EHF) as a first-line treatment, and amino acid-based formula (AAF) should be used in severe clinical cases or when there is a lack of response to EHF. These formulas are nutritionally regulated and fortified; thus, if consumed in adequate amounts, they should cover an infant’s macro- and micronutrient requirements. The nutritional risk of a cow’s milk protein-free diet usually arises once infants or toddlers reduce consumption of their mothers’ milk or formula and start complementary feeding, often with dietary restrictions. At this stage, particular attention should be paid to energy density and meal volume, as young children have relatively high nutrient requirements in relation to their gastric capacity and may not be able to compensate for energy density reduction by only increasing food volume. If not accompanied by proper nutritional counseling, addressing age- and weight-appropriate energy and nutrient requirements, a cow’s milk protein-free diet may increase the risk of micro- and macronutrient deficiencies. Calcium and vitamin D inadequacy are the most frequently reported concerns, but low consumption of iron, zinc, iodine and vitamin B12 have also been described. Inadequate intake of calcium and vitamin D may impair bone mineralization and skeletal development, while inadequate energy and protein intake may contribute to compromised linear growth. Therefore, nutritional management should include regular monitoring of weight, length/height and growth trajectory, particularly in children with pre-existing growth faltering or multiple dietary restrictions. For these reasons, it is vital that, if ordered, a cow’s milk protein-free diet should be followed by dietary counseling and supplementation, but more importantly, it should be time-limited and accompanied by allergen reintroduction and clinical reassessment to reduce unnecessary dietary restrictions [7,38,43].

In more severe cases, when a cow’s milk protein-free diet does not improve symptoms, escalation to a multi-food elimination diet is often empirically prescribed, excluding products such as egg, soy, wheat or other common foods, thus increasing nutritional risks. Each excluded product significantly reduces diet variety and may compromise energy and macro- and micronutrient intake, particularly when excluded foods are replaced by alternatives with lower energy or nutritional density, potentially aggravating pre-existing growth faltering. The number and nutritive importance of the removed foods, as well as the acceptability and quality of the proposed replacements, will impact the nutritional risk of a multi-food elimination diet. On the other hand, available data indicates that in severe cases of FA, even a very restrictive elimination diet can be nutritionally safe if followed by individualized, professional dietary counseling and medical assessment of growth and symptoms by a multidisciplinary team [5,6,44,45]. The nutritional considerations during elimination diets according to feeding stage and extent of dietary restriction are summarized in Table 5.

Elimination diets, especially those removing several foods, can impact not only the nutritional safety of an infant or toddler but also influence eating habits by narrowing the child’s sensory and experimental exposure to tastes and textures in the critical period of acquiring oral motor skills, thus compromising texture acceptance and dietary variety. Repeated and prolonged food removal, as well as unpleasant gastrointestinal symptoms, such as regurgitation, abdominal distention or compromised stooling patterns, that are associated with eating, together with parent anxiety related to allergen introduction, can contribute to the development of neophobia and avoidance of products that are, in fact, tolerated. In infants and toddlers, these present as food/bottle refusal, feeding difficulties, slow eating, selective eating and reduced enjoyment of meals, whereas in older children, they can progress or contribute to avoidant/restrictive food intake disorder (ARFID). The risk appears to be greater in prolonged elimination diets and multi-food restrictions, emphasizing the importance of timely reintroduction of tolerated food as well as responsive feeding guidance, feeding therapy and dietary supervision [46,47,48].

In addition to nutritional risks, elimination diets may place a considerable psychological and financial burden on the child and its family, substantially impacting quality of life. The constant need to monitor food ingredient labels, vigilance for allergen meal content or cross-contact, and eating restrictions in nurseries, schools, restaurants or while traveling can compromise dietary spontaneity and lead to social contact avoidance. The feeling of being different and excluded from peer activities can cause frustration or even depression in a child, while parents cope with chronic anxiety related to meal planning, avoidance of accidental exposure and reactions or entrusting feeding to relatives or childcare providers. These problems are amplified with multi-food restrictions and prolonged elimination. Financial burdens related to these dietary restrictions consist of hypoallergenic formulas, allergen-free and fortified replacement foods, dietetic consultations and medication costs [8,48,49,50]. In a US population-based analysis, family-borne costs accounted for most of the estimated economic burden of childhood allergy, and specialized food represented 31% of out-of-pocket expenditure [50]. Although absolute costs and reimbursements vary across healthcare systems, this illustrates the economic relevance of allergen-restrictive diets.

## 8. Clinical Decision-Making in Differentiating DGBI and FA

Gastrointestinal symptoms such as regurgitation, excessive crying, abdominal distention or altered stooling patterns are common in early childhood, and in a thriving infant or toddler are usually a presentation of a DGBI rather than an FA. That is why the first line of clinical assessment should concentrate on growth pattern evaluation, feeding habits, symptom chronology, general condition and the presence of alarm features. In the absence of the latter and with normal linear growth, the focus should be placed on feeding practices, including type of feeding—breastfed or formula—type of formula, volume and frequency of feeds, and adequate feeding position or accessories used. In particular, first-time parents of newborns and small infants require reassurance and a clear explanation of normal development and physiological processes. More frequent follow-up visits are advisable in order to assess response to feeding habit modification and to identify emerging alarm features such as faltering growth, persistent vomiting, and rectal bleeding, which warrant further evaluation for possible organic diseases, including FA.

FA should be taken into consideration when symptoms are reproducibly associated with specific food ingestion, especially if associated with manifestations from other systems such as cutaneous, respiratory or systemic reactions. A time-limited diagnostic elimination diet, adjusted to the suspected condition, should be undertaken under clinical and dietary supervision, followed by planned allergen reintroduction or oral food challenge to confirm the diagnosis. In the absence of a clinically meaningful response, the elimination diet should be discontinued and alternative diagnoses should be considered, thereby avoiding prolonged unnecessary restriction, potential nutritional inadequacy, and delay of an appropriate etiology-directed management [7,8,38]. The practical decision points for clinicians are presented in Table 6. We also propose a clinical algorithm for rational use of elimination diets in infants and toddlers with gastrointestinal symptoms, which is presented in Figure 1.

## 9. Evidence Gaps and Future Research

Sound evidence for the use of elimination diets in infants and toddlers with DGBI is lacking. The studies described in the literature are fragmented, age-dependent and consist of small cohorts, despite the fact that this age group accounts for the majority of clinical dietary trials in this field. The most pressing research need is double-blind, placebo-controlled RCTs enrolling larger cohorts of infants and toddlers with DGBI that use standardized entry points in accordance with Rome V Criteria, thus enabling proper comparison between studies and drawing evidence-based conclusions. Another research gap is the lack of predictive biomarkers that could easily and repeatedly identify children with non-IgE-mediated allergies or those who could benefit from elimination diets and distinguish them from those who are experiencing functional, age-dependent symptoms. Finally, studies focusing on the long-term effects of elimination diets in DGBI are lacking, and future studies should follow growth patterns, nutritional status or feeding skill development, as well as identify children with specific DGBI that may benefit from some sort of dietary restriction.

## 10. Conclusions

In infants and toddlers, DGBI are the most common reason for clinical symptoms such as regurgitation, infantile distress syndrome, abdominal distention or stooling pattern change, all of which can also be representative of FA. This is why, in order to relieve a child’s symptoms and address parent anxiety, elimination diets are often prescribed, a cow’s milk protein-free diet being the first and most common. Available research data, though, does not support this line of intervention; rather, it suggests avoiding routine empirical elimination for isolated functional gastrointestinal symptoms and highlights that unnecessary restrictions can do more harm than good in this age group, especially by compromising linear growth, causing nutritional deficiencies or promoting poor feeding skills. It is crucial to highlight that if clinical alarm features are present and an infant or toddler is suspected of having an FA, implemented elimination diets should be time-limited and followed by planned allergen reintroduction, as well as supported by professional dietary care. In this narrative review, we tried to show that there is still a big research gap in identifying infants and toddlers with DGBI who will truly benefit from elimination diets, with no sound biomarker or clear algorithm to guide clinicians, thus often leading to FA overdiagnosis, unnecessary dietary restrictions, and their consequences. We also tried, given the limited evidence, to propose a practical algorithm for clinicians caring for infants and toddlers with DGBI and/or suspected FA to improve everyday practice.

## Figures and Tables

**Figure 1:**
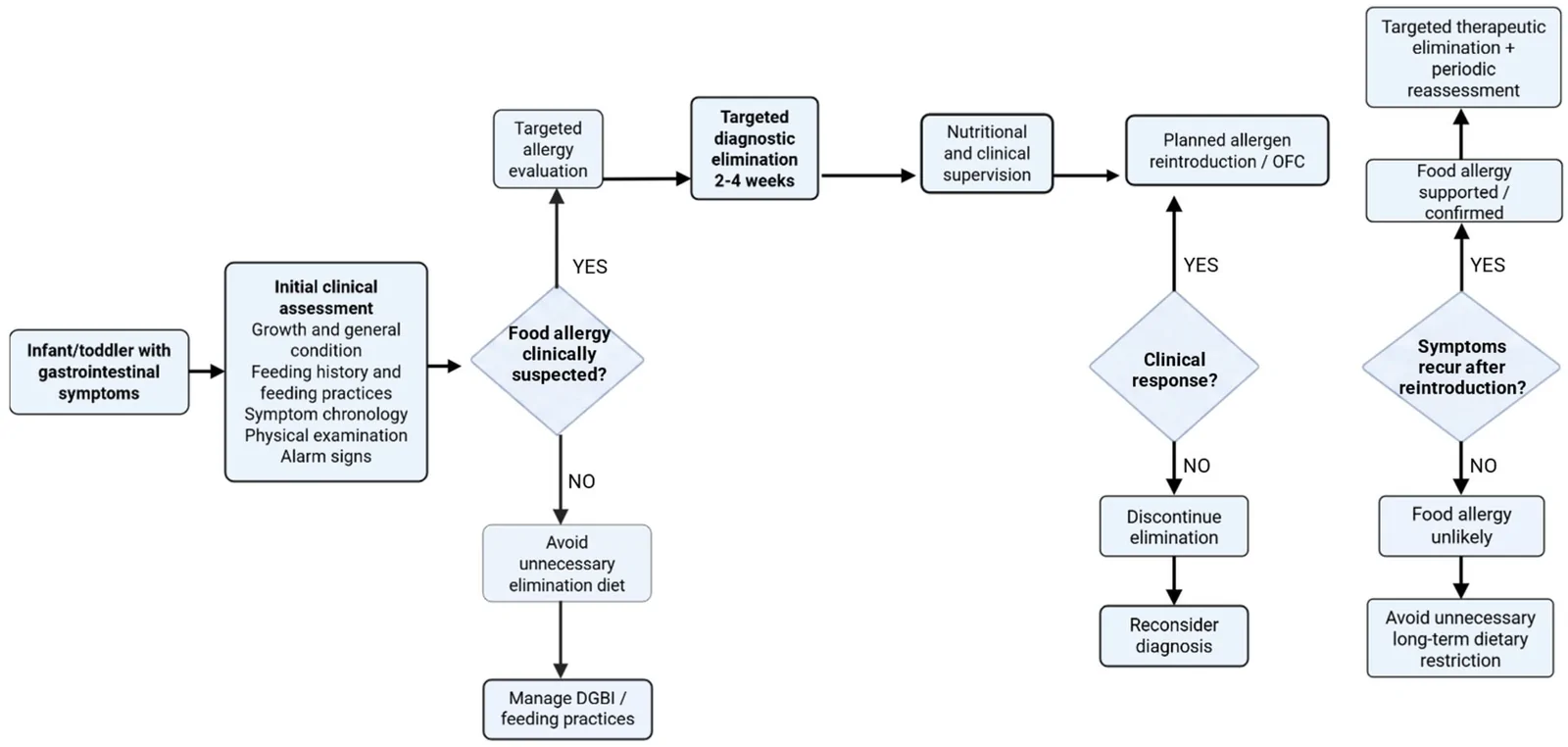
Clinical algorithm for rational use of elimination diets in infants and toddlers with gastrointestinal symptoms.

**Table 1:** Summary of alarm features suggestive of organic disorders rather than DGBI.

Clinical Domain	Alarm Features	Differential Diagnosis
Growth and general condition	Faltering growth, weight loss, poor weight gain, lethargy, fever, or marked irritability/inconsolable pain	Inadequate intake, malabsorption, chronic infection or inflammation, food allergy, endocrine/metabolic disease
Vomiting	Bilious, persistent forceful/projectile, nocturnal vomiting, or hematemesis	Intestinal obstruction or malrotation; hypertrophic pyloric stenosis; raised intracranial pressure; gastroesophageal reflux disease, eosinophilic esophagitis; upper gastrointestinal bleeding
Stool characteristics	Rectal bleeding, melaena, chronic diarrhea, steatorrhea, or delayed passage of meconium	Food protein-induced allergic proctocolitis or enteropathy, gastrointestinal infection, inflammatory bowel disease, malabsorption, Hirschsprung disease
Abdominal findings	Significant or progressive abdominal distension, localized tenderness, guarding, palpable mass, hepatomegaly, or splenomegaly	Intestinal obstruction, severe constipation, inflammatory or acute abdominal disease, hepatobiliary disease, abdominal mass
Feeding and swallowing	Dysphagia, odynophagia, food impaction, recurrent choking/coughing during feeds, persistent feeding refusal, or inadequate oral intake	Structural esophageal abnormality, eosinophilic esophagitis, aspiration, neuromuscular dysfunction, painful esophagitis
Neurological features	Structural esophageal abnormality, eosinophilic esophagitis, aspiration, neuromuscular dysfunction, painful esophagitis	Raised intracranial pressure, central nervous system pathology, neuromuscular or metabolic disorder
Allergy-related indicators	Urticaria, angioedema, wheezing, anaphylaxis, or recurrent gastrointestinal symptoms temporally and reproducibly related to ingestion of a specific food; severe persistent eczema	IgE-mediated food allergy; non-IgE-mediated food allergy; eosinophilic gastrointestinal disease
Clinical course	Late onset, progressive symptoms, persistence beyond the expected age range, or failure to respond to appropriate first-line management	Organic gastrointestinal disorder, infection, food allergy, anatomical abnormality, metabolic or neurological disease

**Table 2:** Immediate and non-immediate reactions and clinical manifestations of food allergies.

	Immediate Reactions	Non-Immediate or Delayed Reaction
Onset of symptoms	Minutes to hours	Days to weeks
Mechanism	IgE-mediated, involves mast cells and histamine	Non-mediated by Ig-E, heterogeneous and incompletely understood
Organ/system		
Skin	Pruritus; erythema Acute urticaria Angioedema Exacerbation of atopic dermatitis	Pruritus Erythema Atopic dermatitis
Gastrointestinal tract	Angioedema of the lips, tongue, and palate Dysphagia Crampy abdominal pain Acute vomiting and diarrhea Bleeding	Vomiting/GERD Food aversion Loose or frequent stools Bloody stools Perianal erythema Constipation Abdominal distension
Respiratory tract	Sneezing; watery rhinorrhea Nasal obstruction Stridor/wheezing/cough Asthma attack Dyspnea	Nasal obstruction Cough
Systemic reaction	Anaphylaxis Hypotonia Signs of hypovolemic shock with metabolic acidosis, vomiting, and diarrhea	Irritability Failure to thrive Anemia
Specific disorders	FPIES	FPIAP FPE

FPIES—food protein-induced enterocolitis syndrome, FPIAP—food protein-induced allergic proctocolitis, FPE—food protein-induced enteropathy, GERD—gastroesophageal reflux disease.

**Table 3:** Clinical situations in which elimination diets are recommended according to current international guidelines.

Clinical Situation	Role of Elimination Diet	Recommendation	Supporting Guideline(s)
Suspected non-IgE-mediated CMA	Diagnostic elimination (2–4 weeks) followed by allergen reintroduction	Recommended	ESPGHAN 2024 [7]; WAO DRACMA 2023 [38]
IgE-mediated food allergy	Targeted elimination of confirmed allergen	Recommended	EAACI 2025 [8]
FPIAP	Maternal cow’s milk elimination (breastfed infants) or extensively hydrolyzed formula (formula-fed infants	Recommended	ESPGHAN 2024 [7]; WAO DRACMA 2024 [38]
Confirmed food allergy	Therapeutic elimination with periodic reassessment	Recommended	EAACI 2025 [8]; ESPGHAN 2024 [7]

CMA—cow’s milk allergy; FPIAP—food protein-induced allergic proctocolitis; ESPGHAN—European Society for Pediatric Gastroenterology, Hepatology and Nutrition; WAO—World Allergy Organization; EAACI—European Academy of Allergy and Clinical Immunology.

**Table 4:** Summary of published evidence and clinical implications for elimination diets in infants with disorders of gut–brain interaction.

Condition	Available Evidence	Interpretation	Practical Implication	Key References
Infant Distress Syndrome (IDS)	Several RCTs and one systematic review with inconsistent findings; reported benefits mainly in infants with suspected concomitant CMA	Benefit cannot be confirmed in unselected infants	Consider only if CMA is clinically suspected	[1,2,5,7,40]
Regurgitation	Few clinical studies, largely involving infants with suspected CMA.	Routine elimination diets are not supported in isolated regurgitation	Optimize conservative management before considering dietary elimination	[1,2,7,41]
Functional constipation	RCTs and observational studies with conflicting findings, mainly in refractory constipation with suspected CMA	Benefit remains uncertain	Consider only in selected refractory cases with suspected CMA	[5,7,16,42]
Infant dyschezia	No intervention studies evaluating elimination diets identified	No evidence supporting routine elimination diets	Reassurance and parental education remain the cornerstone of management	[1,2]
Functional diarrhea	No intervention studies evaluating elimination diets identified	No evidence supporting routine elimination diets	Avoid unnecessary dietary restriction unless a food allergy is clinically suspected	[1,2,5,7,40]

IDS—Infant Distress Syndrome; CMA—cow’s milk allergy; RCT—randomized controlled trial.

**Table 5:** Nutritional considerations during elimination diets according to feeding stage and extent of dietary restriction.

Feeding Stage	Main Nutritional Risks During Elimination Diets	Recommended Nutritional Management
Exclusively breastfed infant	Maternal elimination diets may reduce maternal intake of calcium, vitamin D and iodine, with potential implications for maternal nutritional status if supplementation is inadequate	Ensure maternal dietary counseling and appropriate supplementation (particularly calcium, vitamin D and iodine); reassess the indication for maternal elimination regularly
Formula-fed infant	Inappropriate formula choice or prolonged use of nutritionally inadequate milk substitutes may compromise protein, energy and micronutrient intake	Use an extensively hydrolyzed formula (EHF) or amino acid formula (AAF) when indicated; monitor growth and nutritional adequacy
Complementary feeding (6–12 months)	Elimination of cow’s milk, egg or wheat may reduce intake of protein, calcium, iron, zinc, iodine, vitamin D and selected B vitamins at a period of rapid growth	Provide appropriate food substitutions, monitor complementary feeding practices and assess growth and nutrient intake
Toddler (>12 months)	Prolonged or multiple food elimination diets increase the risk of inadequate energy, protein and micronutrient intake, selective eating and impaired dietary diversity	Individualize dietary management, encourage dietary diversity, monitor growth and nutritional status, and periodically reassess the need for continued elimination
Multiple food elimination (any age)	The risk of macro- and micronutrient deficiencies increases with the number of excluded foods and the duration of dietary restriction	Dietitian supervision is strongly recommended, together with regular nutritional assessment and planned food reintroduction whenever possible

EHF—extensively hydrolyzed formula, AAF—amino acid formula.

**Table 6:** Decision points for clinicians in differentiating DGBI and FA.

Clinical Feature	DGBIs More Likely	FAs More Likely
Growth and general condition	Normal growth and thriving	Faltering growth, dehydration, enteropathy, systemic illness
Symptom pattern	Age-dependent, fluctuating, often related to feeding volume, bowel habits, or developmental stage	Reproducible reaction after a specific food exposure
Extraintestinal manifestations	Usually absent	Urticaria, angioedema, eczema flare, wheezing, pallor, lethargy
Regurgitation or colic alone	Common and usually functional	Insufficient alone to diagnose CMA
Response to elimination	May improve spontaneously or due to nonspecific changes	Improvement during elimination and recurrence on reintroduction

DGBI—disorders of gut–brain interaction; FA—food allergy; CMA—cow’s milk allergy.

## Data Availability

No new data were created or analyzed in this study. Data sharing is not applicable to this article.

## Abbreviations

The following abbreviations are used in this manuscript:

DGBI	Disorders of gut–brain interaction
FA	Food allergy
CMA	Cow’s milk allergy
IDS	Infant Distress Syndrome
FC	Functional constipation
OFC	Oral food challenge
FPIES	Food protein-induced enterocolitis syndrome
FPIAP	Food protein-induced allergic proctocolitis
FPE	Food protein-induced enteropathy
ESPGHAN	European Society for Pediatric Gastroenterology, Hepatology and Nutrition
EAACI	European Academy of Allergy and Clinical Immunology
EHF	Extensively hydrolyzed formula
AAF	Amino acid-based formula
ARFID	Avoidant/restrictive food intake disorder
RCT	Randomized controlled trial
